# Overexpression of Notch2 enhances radiosensitivity via inhibition of the AKT/mTOR signaling pathway in nasopharyngeal carcinoma

**DOI:** 10.1080/21655979.2021.1949236

**Published:** 2021-07-05

**Authors:** Li-Zhi Wu, Mao-Ling Huang, Cheng-Lin Qi, Li-Jun Shen, You Zou, Rui Yang, Jian-Fei Sheng, Shi-Ming Chen

**Affiliations:** a Department of Otolaryngology-Head and Neck Surgery, Renmin Hospital of Wuhan University, Wuhan, P. R. China; b Research Institute of Otolaryngology-Head and Neck Surgery, Renmin Hospital of Wuhan University, Wuhan, P. R. China

**Keywords:** Nasopharyngeal carcinoma, Notch2, radiosensitivity, AKT/mTOR signaling pathway

## Abstract

Our previous study found that in nasopharyngeal carcinoma (NPC) cells, overexpression of Notch2 can inhibit epithelial-mesenchymal transition (EMT), which plays a vital role in mediating radiosensitivity. The purpose of this study was to explore the radiosensitizing efficacy of the Notch2 gene in NPC cells and its potential mechanism. We used the recombinant plasmid transfection technique to establish Notch2-overexpressing 5–8 F and CNE-2 NPC cells. Cell proliferation, radiosensitivity, apoptosis and cell cycle distribution were assessed by cell counting kit-8 (CCK-8) experiments, colony formation experiments and flow cytometry. The levels of proteins related to cell cycle, apoptosis, and the AKT/mTOR signaling pathway were evaluated by using Western blotting. The results suggested that Notch2 overexpression increased the radiosensitivity of NPC cells, with sensitizing enhancement ratios (SERs) of 1.24 (5–8 F cells) and 1.34 (CNE-2 cells). Flow cytometry indicated that the level of apoptosis and percentage of cells in G2/M-phase were highest in NPC cells overexpressing Notch2 and treated with radiotherapy compared to cells overexpressing Notch2 alone or administered radiotherapy alone. Western blotting showed that compared to that of cells treated with Notch2 overexpression or radiotherapy alone, the levels of γH2AX, Bax, Bcl-2, Cyclin D1 and AKT/mTOR signaling pathway-related proteins were modified in NPC cells overexpressing Notch2 and treated with radiotherapy. These findings showed that overexpression of Notch2 can increase the radiosensitivity of NPC cells by inhibiting the AKT/mTOR pathway.

Abbreviations

NPC: Nasopharyngeal carcinoma; EMT: Epithelial-mesenchymal transition; CCK8: Cell counting kit-8; EBV: Epstein-Barr virus; FBS: Fetal bovine serum; PE: Plating efficiency; SF: Survival fraction; SER: Sensitizing enhancement ratio; DSBs: DNA double-strand breaks

**Figure:**
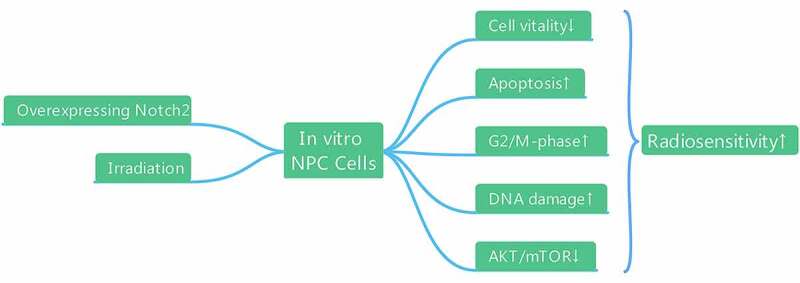

## Introduction

Nasopharyngeal carcinoma (NPC) is a malignant neoplasm originating from the epithelium of the nasopharyngeal mucosa, and risk factors include Epstein-Barr virus (EBV) infection, dietary habits, and genetic predisposition [1]. According to an epidemiological survey, there were approximately 129,000 patients with newly diagnosed NPC in 2018, with an extremely unbalanced geographical distribution: nearly 70% of new cases occurred in East Asia [2]. Radiotherapy is the preferred treatment for patients with NPC; however, there are still some NPC patients who develop local recurrence and have a poor prognosis due to radiation resistance [3,4]. Therefore, exploring new therapeutic strategies to enhance the radiosensitivity of NPC is essential in improving the curative effect of NPC radiotherapy and the prognosis of NPC.

AKT/mTOR is a signaling pathway that regulates proliferation, apoptosis, and the distribution of the cell cycle in tumor cells, and the excessive activation of this signaling pathway promotes radioresistance in various tumors [5]. Many studies have found that inhibiting the AKT/mTOR signaling pathway can either increase radiation-induced apoptosis or repress the repair of DNA double-strand breaks (DSBs) to achieve a radiosensitizing effect [6,7].

The Notch2 gene is located on human chromosome 1p12 (1:119,911,553–120,069,703) and is composed of 34 exons, encoding a total of 2471 amino acids. Notch2 is highly expressed in hepatocellular carcinoma [8] and non-small-cell lung cancer [9], promotes proliferation of stem-like tumor cells and increases cell resistance to radiotherapy. In contrast, Wang et al. examined the protein level of Notch2 in tumor and adjacent normal tissues from 184 colorectal cancer patients and found that the level of Notch2 protein was lower in tumor tissues than in adjacent normal tissues, suggesting that low expression of Notch2 protein might be an indicator of poor prognosis in colorectal cancer [10]. In addition, our previous study also found that overexpression of Notch2 can inhibit epithelial-mesenchymal transition (EMT) in NPC cells [11]. Many studies have confirmed that EMT plays a vital role in mediating the radioresistance of tumor cells and inhibiting EMT could increase the radiosensitivity of various types of carcinoma, such as non-small-cell lung carcinoma, hepatocellular carcinoma, gastric carcinoma and breast carcinoma [12].

Based on this consensus, we hypothesized that overexpression of Notch2 might enhance the radiosensitivity of NPC cells. To address this, we generated Notch2-overexpressing 5–8 F and CNE-2 NPC cells to investigate the efficacy of Notch2 overexpression combined with radiation on cell proliferation, apoptosis, cell cycle progression and the AKT/mTOR signaling pathway with the aim of exploring the underlying mechanisms by which Notch2 overexpression potentially increases the radiosensitivity of NPC cells and providing new ideas for therapeutic strategies for NPC.

## Materials and methods

### Cell culture and transfection

The 5–8 F and CNE-2 NPC cell lines (China Center for Type Culture Collection, Wuhan, China) were cultured in RPMI 1640 medium (Jenom, Hangzhou, China) supplemented with 10% fetal bovine serum (FBS) (Gibco; Thermo Fisher Scientific, MA, USA) and maintained in a cell incubator.

The recombinant plasmid H9511 containing Notch2 cDNA was purchased from HeYuan Biotechnology (Shanghai, China; https://www.obiosh.com). The lentivirus transfection packaging plasmid system was configured with the following reagents: 7.5 µg pSPAX2, 5 µg pMD2.G, 10 µg plasmid H9511, 50 µl of Lipofectamine 2000 (Invitrogen, CA, USA) and 250 µl of serum-free culture medium; these reagents were combined for to transfect 293 T cells, and the viral supernatant was collected 48 h later. NPC cells were infected with the virus from the supernatant collected above. After 48 h of infection, complete medium containing 5 μg/ml puromycin (Sigma, St Louis, USA) was added to screen the cells for 14 days [13].

### Cellular irradiation

The cells were irradiated vertically at doses of 2, 4, and 6 Gy at room temperature using a 6 MV X-ray Varian linear accelerator [14].

### Experimental groups

We prepared four treatment groups: control group (5–8 F or CNE-2 cells); oeNotch2 group (Notch2-overexpressing 5–8 F or CNE-2 cells); IR+control group (6 Gy-irradiated 5–8 F or CNE-2 cells); and IR+oeNotch2 group (Notch2-overexpressing 5–8 F or CNE-2 cells subjected to 6 Gy of radiation).

### Cell proliferative activity assay

The four groups of cells were trypsinized and then seeded into 96-well culture plates at a density of 5 × 10^3^ cells in each well, with three replicate wells per group. At 24 h, 48 h and 72 h after seeding, 10 µl of CCK8 reagent (Dojindo, Japan) was added to each well, and the plates were incubated for 1 h. The optical density of each well was measured at 450 nm by a Victor3 microplate reader (Perkin-Elmer, Waltham, MA, USA) [15].

### Colony formation assay

We prepared control and oeNotch2 cells and subjected them to different doses of radiation. After 10 days of culture, the cells were fixed with methanol for 10 min, stained with 1% crystalline violet reagent, and washed with PBS (Jenom, Hangzhou, China). Colonies containing more than 50 cells were counted. The plating efficiency (PE) was calculated as the number of colonies/planted cells×100%, and the survival fraction (SF) was calculated as the irradiated colony formation rate/unirradiated colony formation rate×100%. The single-hit multitarget model was used to fit the cell survival curve, and the mean lethal dose (D0), the quasi-threshold dose (Dq), and the sensitizing enhancement ratio (SER) values were calculated with Prism 8 statistical software [16].

### Cell cycle assay

The 4 treatment groups were established as previously described, incubated for 24 h, and digested with EDTA-free trypsin (Jenom, Hangzhou, China) to prepare single-cell suspensions. After the cells were centrifuged, they were resuspended in a solution comprising 200 μl of Triton, 100 μl of RNase, and 10 μl of PI (MultiSciences, China), incubated for 20 min without light, and then filtered through a 300-mesh nylon filter. The fluorescence intensity of the PI-DNA complexes in cells was measured with a FACS Calibur flow cytometer (BD Biosciences, NJ, USA), and cell cycle distribution was calculated by using ModFitLT V3.1 [17].

### Apoptosis assay

The 4 treatment groups were established as previously described, incubated for 24 h, and digested with EDTA-free trypsin (Jenom, Hangzhou, China) to prepare single-cell suspensions. After the cells were centrifuged, they were stained with Annexin V-APC and 7-AAD, incubated for 30 min without light, and then filtered through a 300-mesh nylon filter. Apoptotic cells were evaluated on a FACS Calibur flow cytometer (BD Biosciences, NJ, USA), and analysis of the experimental data was carried out by using FlowJo software [18].

### Western blot analysis

As described previously, we extracted proteins from cells with RIPA lysis buffer, separated equal amounts of protein using SDS-PAGE, and then transferred the protein to a 0.45-µm PVDF membrane (Millipore, USA). Next, the membrane was blocked with 5% skim milk for 1 h at room temperature before it was washed 3 times with 1× TBST for 10 min each time. The membranes were then incubated with the following antibodies: rabbit anti-GAPDH (#5174), anti-Notch2 (#5732), anti-Bax (#0120), anti-Bcl-2 (#6139), anti-γH2AX (#8482), anti-Cyclin D1 (#0931), anti-AKT (#6261), anti-phospho-AKT (#0016), anti-mTOR (#6308), and anti-phospho-mTOR (#3308), all of which were purchased from Affinity Biosciences (OH, USA). A chemiluminescence reagent was used to visualize the protein bands, and the grayscale values of the bands were measured with Image Lab 6.0 software (Bio-Rad) [19].

### Statistical analysis

All experiments were repeated independently 3 times. Experimental data are presented as the means±SD and were analyzed by using Prism 8 statistical software (GraphPad, USA). Comparisons between the two independent samples were conducted by Student’s t-test. A P-value <0.05 was considered statistically significant.

## Results

In this study, we hypothesized that overexpression of Notch2 could enhance the radiosensitivity of NPC cells. Therefore, we established NPC cells with Notch2 overexpression using a lentiviral transfection technique and found that overexpression of Notch2 could improve the radiosensitivity of NPC cells in vitro by inhibiting cell proliferation, reducing viability, increasing G2/M-phase arrest, promoting apoptosis, and inhibiting the AKT/mTOR signaling pathway.

### Confirmation of Notch2 overexpression in NPC cells

**Figure 1.**
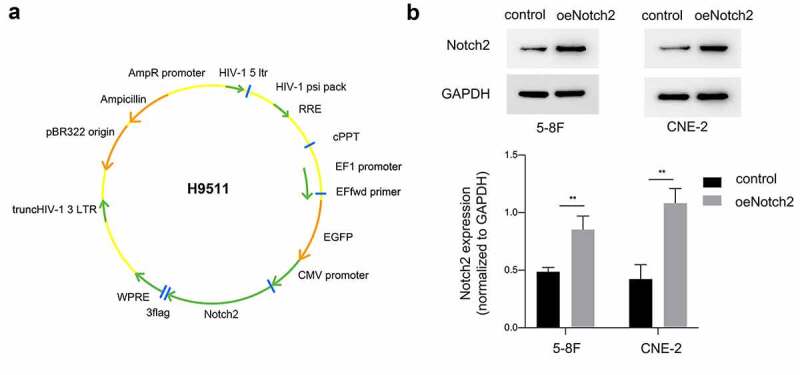
Confirmation of Notch2 overexpression in NPC cells. (a) Schematic of the recombinant plasmid H9511 containing Notch2 cDNA. (b) Relative protein expression of Notch2 in the oeNotch2 group compared to the control group. The plotted data are shown as the means±SD of three independent experiments, and the differences between the control and oeNotch2 groups were compared by using Student’s t-test (**P < 0.01)

In this study, we used the recombinant plasmid H9511 (Figure 1(a)) containing Notch2 cDNA to for ectopic expression in 5–8 F and CNE-2 NPC cells. Western blotting was used to detect whether Notch2 protein was overexpressed in NPC cells after infection. As shown in Figure 1(b), the Notch2 protein was significantly overexpressed in the oeNotch2 group compared with the control group, which confirmed that the recombinant plasmid successfully upregulated Notch2 protein expression in 5–8 F and CNE-2 NPC cells.

### Overexpression of Notch2 enhances the radiosensitivity of NPC cells

**Figure 2.**
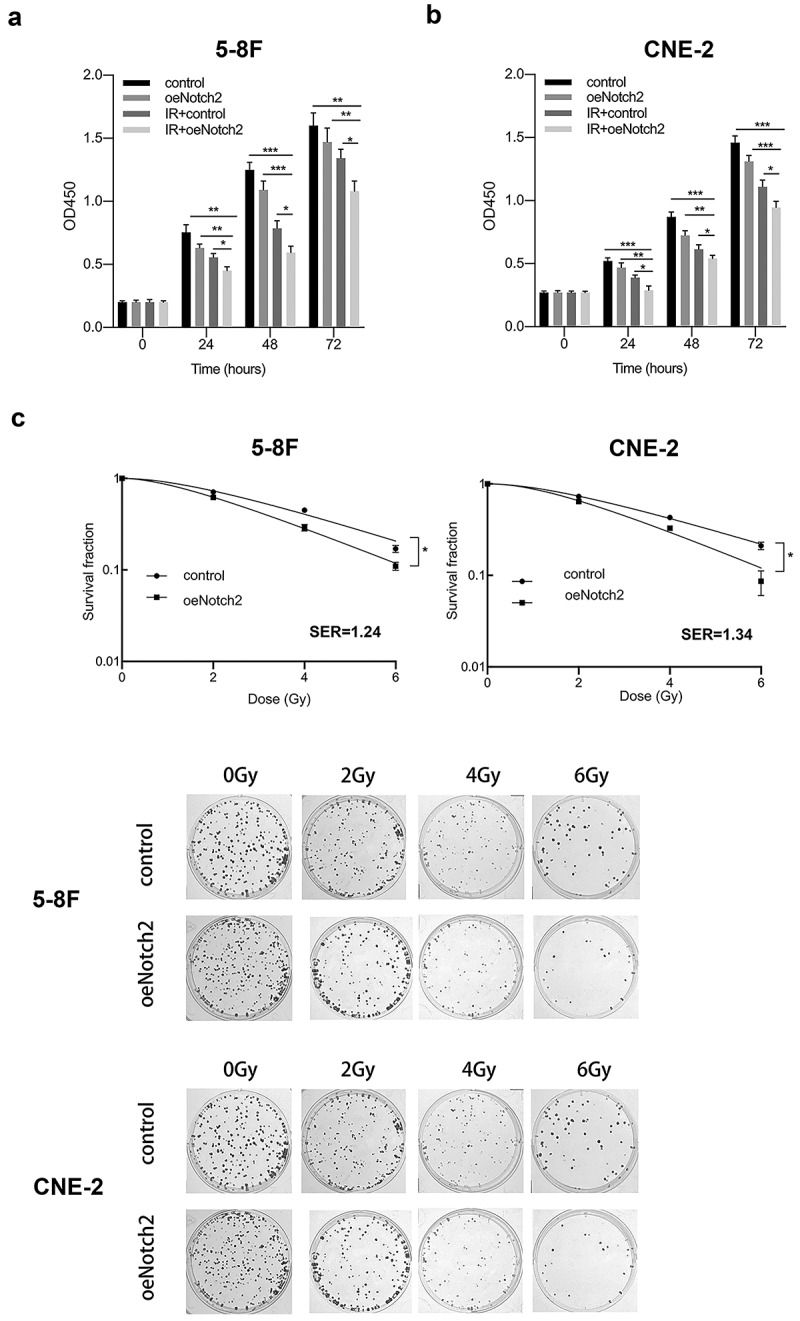
Overexpression of Notch2 enhances the radiosensitivity of NPC cells. (a, b) In 5–8 F and CNE-2 cells, the viability of the control, oeNotch2, IR+control and IR+oeNotch2 groups was detected by the CCK-8 assay. (c) In 5–8 F and CNE-2 cells, the surviving fractions in the oeNotch2 and control groups after radiation at different doses (2, 4, and 6 Gy) are shown. The plotted data are shown as the means±SD of three independent experiments, and the differences between the IR+oeNotch2 and the control, oeNotch2, or IR+control groups were compared by using Student’s t-test. (*P < 0.05; **P < 0.01; ***P < 0.001)

We used CCK-8 assays to measure the proliferative activity of the cells. As shown in Figure 2(a and b), NPC cells with Notch2 overexpression and exposure to radiation showed significantly inhibited proliferation compared to that of NPC cells either treated with radiotherapy alone or only overexpressing Notch2.

To explore whether overexpression of Notch2 has an effect on the radiosensitivity of NPC cells, we exposed the parental and oeNotch2 cells to different doses of radiation. The cell survival curve was plotted according to the colony formation experiment, which suggested that Notch2-overexpressing NPC cells had lower survival fractions than did the respective parental cells radiation (Figure 2(c)). Together, these results suggested that Notch2 overexpression combined with radiation inhibited the survival and vitality of NPC cells more effectively than radiotherapy alone. The SERs of 5–8 F and CNE-2 NPC cells with Notch2 overexpression were 1.24- and 1.34-fold higher, respectively, than that of their parental cell lines as indicated by the radiobiological parameters (Table 1). This finding further confirms that overexpression of Notch2 increases the radiosensitivity of NPC cells.

**Table 1.** Radiobiological parameters fit to the single-hit multitarget model

Cell line	Group	D_0_ (Gy)	D_q_ (Gy)	N	k	SER
5–8 F	IR+control	2.69	1.89	2.02	0.37	1.24
	IR+oeNotch2	2.18	1.41	1.91	0.45	
Cell line	Group	D_0_ (Gy)	D_q_ (Gy)	N	k	SER
CNE-2	IR+control	2.81	1.95	1.90	0.36	1.34
	IR+oeNotch2	2.09	1.68	1.63	0.48	

Abbreviations: D_0_, mean lethal dose; D_q_, quasi-threshold dose; SER, sensitizing enhancement ratio.

### Overexpression of Notch2 increases radiation-induced G2/M-phase arrest and apoptosis

We used flow cytometry to explore whether overexpression of Notch2 has an effect on the cell cycle distribution and apoptosis of NPC cells.

**Figure 3.**
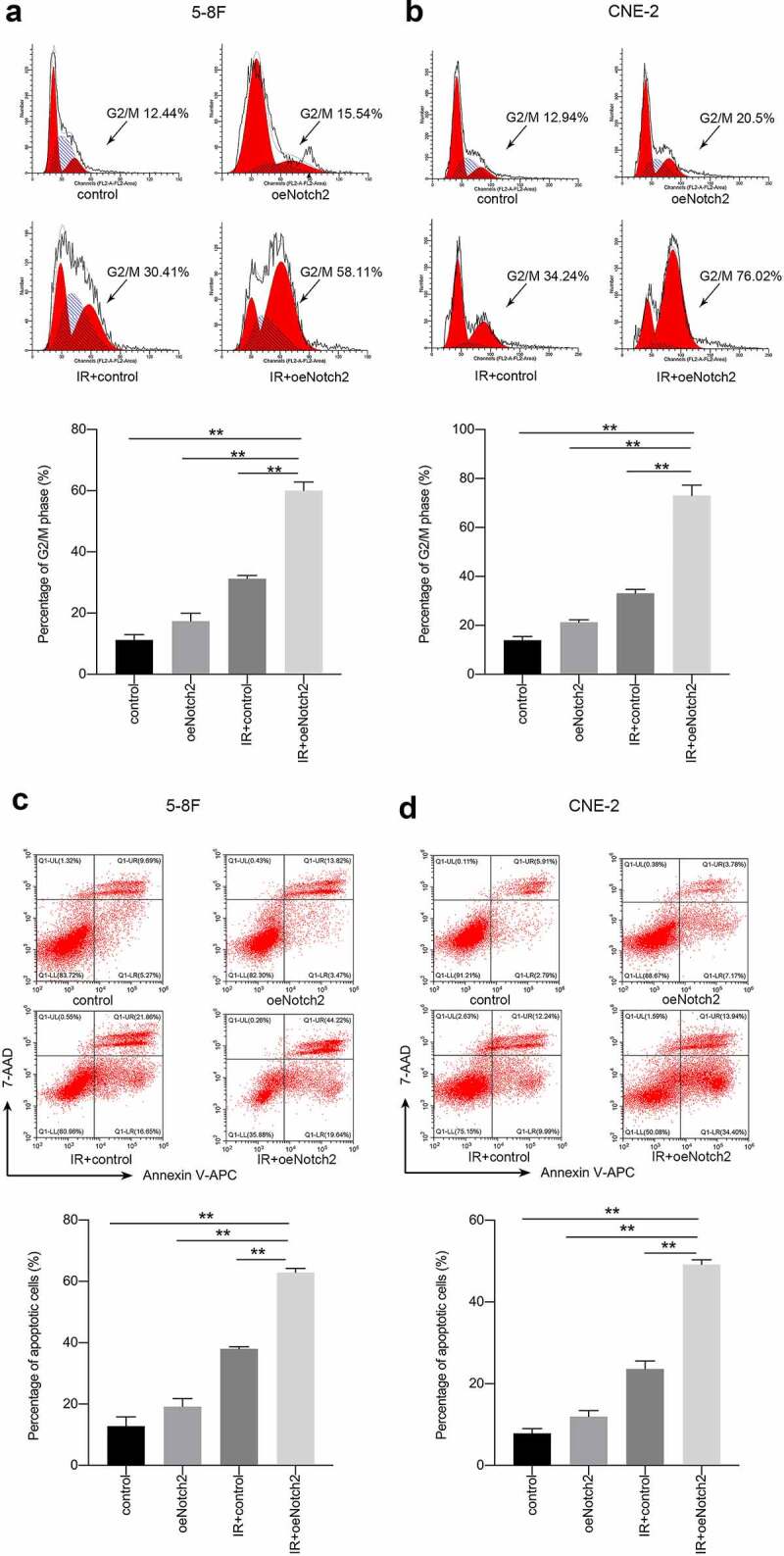
Overexpression of Notch2 enhances radiation-induced G2/M-phase arrest and apoptosis in NPC cells. (a, b) Flow cytometric analysis was performed to detect the percentage of 5–8 F and CNE-2 cells in G2/M phase, which is relatively radiation sensitive. (c, d) Flow cytometric analysis was performed to detect the apoptosis rate in 5–8 F and CNE-2 cells. The plotted data are shown as the means±SD of three independent experiments, and the differences between the IR+oeNotch2 group and the control, oeNotch2, or IR+control groups were compared by using Student’s t-test (**P < 0.01)

The percentage of 5–8 F NPC cells in G2/M phase (a relatively radiation-sensitive phase of the cell cycle) was 17.37 ± 2.58% and 31.21 ± 1.12% after overexpression of Notch2 alone and radiotherapy alone, respectively; however, the percentage of 5–8 F NPC cells in G2/M phase increased to 60.06 ± 2.75% when overexpressing Notch2 and subjected to radiotherapy (Figure 3(a)). Consistent with this finding, the percentage of CNE-2 NPC cells in G2/M-phase was 21.25 ± 1.06% or 33.12 ± 1.58% after overexpression of Notch2 alone or radiotherapy alone, respectively, whereas the percentage increased to 73.01 ± 4.26% in CNE-2 NPC cells overexpressing of Notch2 and subject to radiotherapy (Figure 3(b)).

The apoptosis rate of 5–8 F cells with Notch2 overexpression alone or treated with radiotherapy alone was 19.14 ± 2.62% or 38.02 ± 0.72%, respectively. However, the apoptosis increased to 62.93 ± 1.32% in 5–8 F cells with Notch2 overexpression and subjected to radiotherapy (Figure 3(c)). Consistent with this finding, the apoptosis rate of CNE-2 NPC cells overexpressing Notch2 alone or treated with radiotherapy alone was 11.98 ± 1.45% or 23.62 ± 1.96%, respectively but increased to 49.17 ± 1.17% in CNE-2 NPC cells overexpressing Notch2 and treated with radiotherapy (Figure 3(d)).

In summary, this finding confirmed that Notch2 overexpression increased the percentages of cells in G2/M phase and apoptotic cells.

### Overexpressing Notch2 alters the expression of proteins related to DNA damage, apoptosis and cell cycle progression

**Figure 4.**
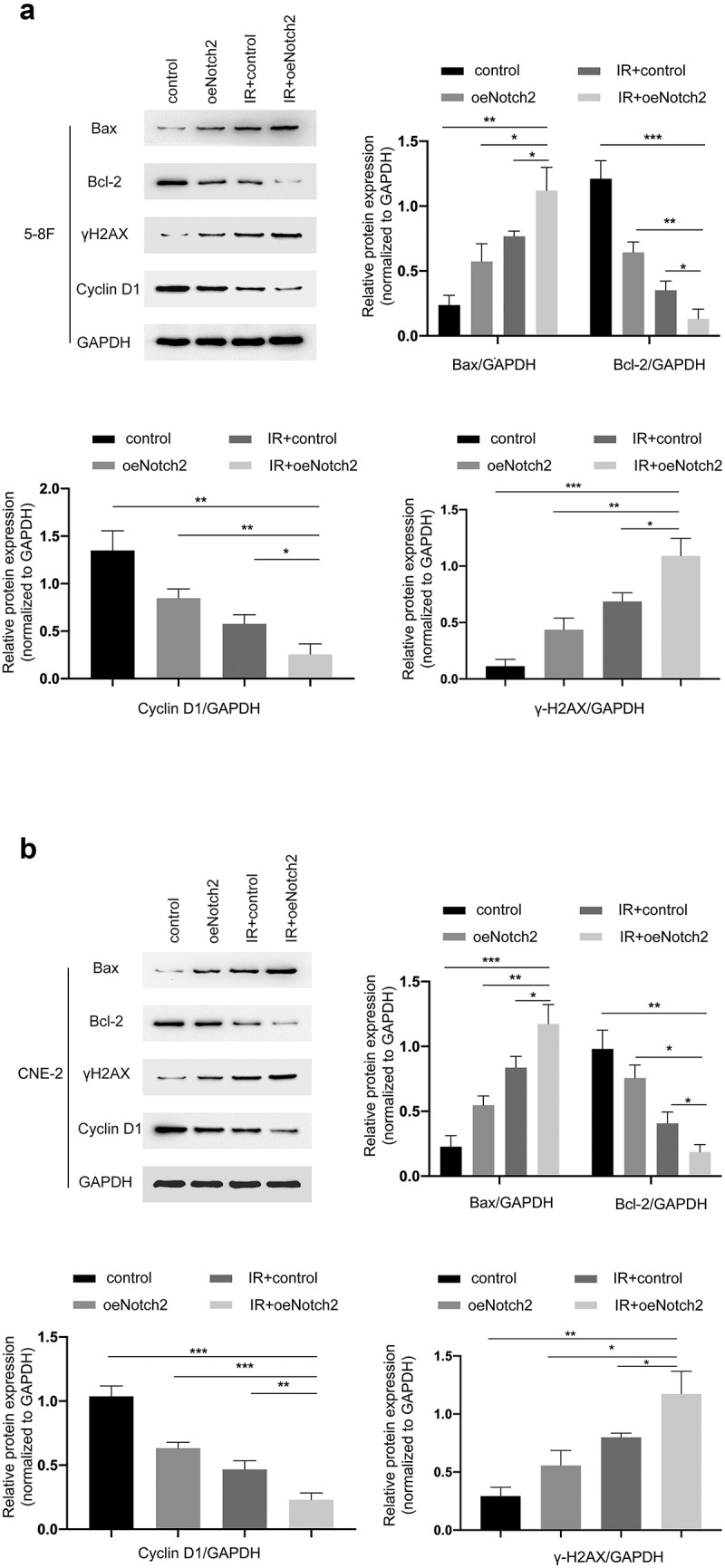
Overexpression of Notch2 alters the expression of proteins related to DNA damage, apoptosis and cell cycle progression in NPC cells. (a, b) Western blot analysis of BAX, Bcl-2, γH2AX and Cyclin D1 expression in the control, oeNotch2, IR+control and IR+oeNotch2 groups. Quantification of BAX, Bcl-2, γH2AX and Cyclin D1 levels is presented as bar graphs. GAPDH was used as a loading control. The plotted data are shown as the means±SD of three independent experiments, and the differences between the IR+oeNotch2 group and the control, oeNotch2, or IR+control groups were compared by using Student’s t-test (*P < 0.05; **P < 0.01; ***P < 0.001)

To further investigate the mechanism by which Notch2 overexpression enhances radiation-induced G2/M-phase blockade and apoptosis, we evaluated the levels of proteins associated with DNA damage, apoptosis, and cell cycle progression using Western blotting. The results showed that in 5–8 F and CNE-2 cells, the expression levels of Bax and γH2AX were higher in the IR+oeNotch2 group than in the control, oeNotch2, and IR+control groups, while the expression levels of Cyclin D1 and Bcl-2 were lower than those in the other three groups (Figure 4(a,b)). These results indicate that compared with radiotherapy alone, overexpression of Notch2 in combination with radiotherapy induced more DNA damage, enhanced the apoptosis of NPC cells by upregulating Bax and promoted G2/M blockade by downregulating the cell cycle protein Cyclin D1, all of which contributed to the enhanced radiosensitivity of NPC cells.

### Overexpression of Notch2 increases the radiosensitivity of NPC cells by inhibiting the AKT/mTOR signaling pathway

**Figure 5.**
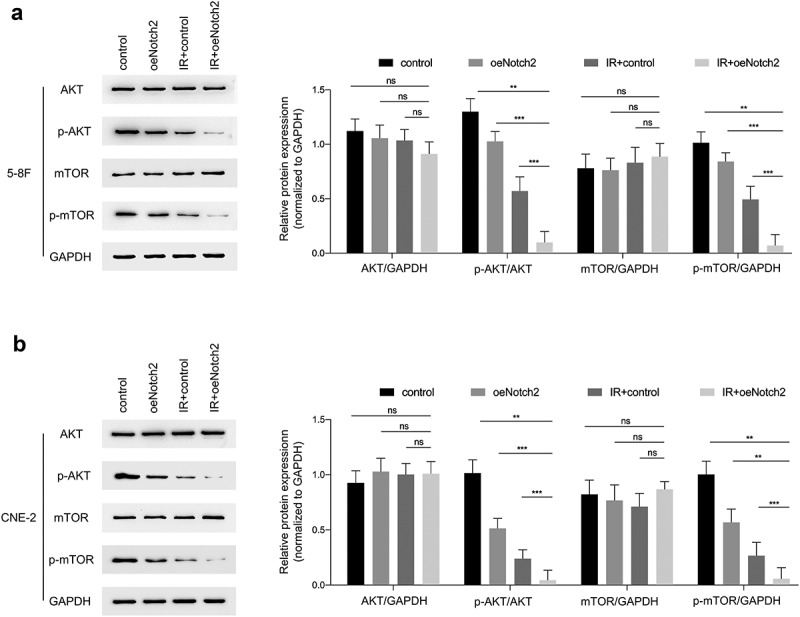
Overexpression of Notch2 inhibits the AKT/mTOR signaling pathway in NPC cells. (a, b) Western blot analysis of AKT, p-AKT, mTOR and p-mTOR expression levels in the control, oeNotch2, IR+control and IR+oeNotch2 groups. Quantification of the AKT, p-AKT, mTOR and p-mTOR is presented in bar graphs. GAPDH was used as a loading control. The plotted data are shown as the means±SD of three independent experiments, and the differences between the IR+oeNotch2 group and the control, oeNotch2. or IR+control groups were compared by using Student’s t-test (ns, P > 0.05; *P < 0.05; **P < 0.01; ***P < 0.001)

It has been reported that AKT/mTOR has a relationship with the radiosensitivity of tumors [20], so we evaluated the effect of Notch2 on this signaling pathway. The results showed that in 5–8 F and CNE-2 cells, the protein expression levels of p-AKT/GAPDH and p-mTOR/GAPDH in the IR+oeNotch2 group were lower than those in the control, oeNotch2, and IR+control groups, there was no change in protein expression of AKT or mTOR after overexpression of Notch2 or irradiation (Figure 5(a,b)).

## Discussion

Radiation therapy is the primary therapy for NPC; however, after radiation therapy, 20–30% of NPC patients still develop local recurrence or have a poor prognosis due to radiotherapy resistance [21]. Therefore, increasing the radiosensitivity of NPC cells has become one of the current directions in NPC treatment.

The colony formation assay is the current gold standard for detecting radiosensitivity, and an SER greater than 1 indicates a sensitization effect [22]. In this study, it was found that after different radiation doses, the proliferation, colony formation capacity and viability of Notch2-overexpressing NPC cells were significantly decreased, with 5–8 F and CNE-2 cells presenting SERs of 1.24 and 1.34, respectively. Overall, Notch2 overexpression was shown to enhance the radiosensitivity of NPC cells. Nie et al. found that after knocking down SALL4, the SER of CNE-2 cells was 1.11 [23], and Zhang et al. found that after knocking down RPA1, the SER of CNE-2 cells was 1.21 [24]. It is obvious that Notch2 has a greater effect on the radiosensitivity of NPC than do other genes and deserves in-depth study.

Radiation induces DNA DSBs by causing direct or indirect damage to cellular DNA. Subsequently, tumor cells activate a number of signaling pathways to initiate DNA damage repair, including the NF-κB, MAPK, AKT/mTOR, and TGF-β signaling pathways. If repair is unsuccessful, cells undergo apoptosis [25]. In the presence of DSBs, histone H2AX is phosphorylated at Ser-139 (γH2AX), which can be used as an indicator of the extent of DNA damage caused by radiation [26]. In this study, we observed that the level of γH2AX was markedly higher in the cells with Notch2 overexpression and treated with radiotherapy than in parental cells treated with radiotherapy, suggesting that Notch2 overexpression enhanced radiation-induced DNA damage.

Cancer cells at different cell cycle phases have different radiosensitivities, and cancer cells in G2/M phase have the highest radiosensitivity [27]. In the present study, we found that Notch2 overexpression combined with radiotherapy resulted in more NPC cells being arrested in G2/M phase. Moreover, the level of Cyclin D1 was significantly reduced in cells with Notch2 overexpression that were treated with radiotherapy than in parental cells treated with radiotherapy. Cyclin D1 plays a vital role in cell cycle progression and tumor progression. Many studies have found that Cyclin D1 promotes the cell cycle transition from G1 to S phase [28], while downregulation of Cyclin D1 can induce G2/M-phase arrest in tumor cells [29]. Thus, overexpression of Notch2 may make NPC cells more sensitive to radiation by downregulating Cyclin D1 expression to increase G2/M-phase arrest.

Bax and Bcl-2 belong to the Bcl-2 family of proteins; Bax promotes apoptosis, while Bcl-2 inhibits apoptosis [30,31]. In the present study, we observed that overexpression of Notch2 significantly increased the expression of Bax and decreased the expression of Bcl-2 in irradiated NPC cells, confirming that Notch2 overexpression enhances radiation-induced apoptosis.

AKT has been proven to play a critical role in the development, migration, invasion, chemoresistance and radioresistance of various malignancies, including NPC. The level of phosphorylated AKT is increased in radioresistant NPC tissues compared to radiosensitive NPC tissues [32]. In addition, the AKT/mTOR pathway is related to radiation resistance in tumor cells and activates not only DNA-PK to enhance DNA damage repair but also NF-κB to inhibit apoptosis [33]. One study showed that the use of dual PI3K/mTOR inhibitors could inhibit the development of NPC and improve its radiosensitivity [34]. Furthermore, celecoxib, an EGFR inhibitor, enhances the radiosensitivity of NPC cells by blocking phosphorylation-mediated activation of the downstream AKT/mTOR pathway [35]. The present study confirmed that, similar to inhibition of EGFR, overexpression of Notch2 blocks phosphorylation-mediated activation of the downstream AKT/mTOR pathway and thus increases the radiosensitivity of NPC cells, suggesting that Notch2 also has a regulatory role in the AKT/mTOR signaling pathway.

Zhang et al. found that overexpression of Circ_0001287 increased the radiosensitivity of non-small-cell lung cancer cells by inhibiting their proliferation, migration, and invasion [36]. Zhou et al. reported that knockdown of survivin increased apoptosis and G2/M-phase arrest and reduced clone formation in cervical cancer cells subjected to radiotherapy [37]. Huang et al. stated that knockdown of MUC1 inhibited the growth of head and neck squamous carcinoma cells and induced apoptosis and subsequently enhanced the radiosensitivity of head and neck squamous carcinoma cells [38]. Despite these discoveries, none of the studies reported radiotherapy sensitization ratios targeting these genes. Therefore, we do not yet know the difference in the sensitivity of targeting these different genes to radiotherapy. The differences in radiotherapy sensitivity of these targets for NPC merit further comparative studies, which will play an important role in translational research for clinical application in the future.

This study has some limitations, the greatest of which is the lack of in vivo experiments. We do not yet know the impact of Notch2 overexpression on the sensitivity to radiation therapy of NPC cells in vivo, and we will investigate this in our future work.

The data from our previous study demonstrated that overexpression of Notch2 could inhibit EMT in NPC cells in vitro and in vivo and that inhibition of EMT could enhance the radiosensitivity of tumor cells. Therefore, we investigated the effects of Notch2 overexpression on the radiosensitivity of NPC cells. In this study, we found that the proliferative activity and colony formation ability of NPC cells were significantly decreased in cells with Notch2 overexpression that were subjected to radiotherapy compared to cells with Notch2 overexpression alone or parental cells subjected to radiotherapy; furthermore, overexpression of Notch2 enhanced radiation-induced G2/M-phase arrest and apoptosis. In addition, our study demonstrates for the first time that Notch2 overexpression enhances the radiosensitivity of NPC cells by inhibiting the AKT/mTOR signaling pathway. Therefore, the Notch2 gene may be an underlying therapeutic target for enhancing radiosensitization in NPC.

## Conclusions

Our study revealed that overexpression of Notch2 could improve the radiosensitivity of NPC cells in vitro by reducing cell viability, increasing G2/M-phase arrest, promoting apoptosis and DNA damage and inhibiting the AKT/mTOR signaling pathway. Therefore, Notch2 may be a new drug target to increase the radiosensitivity of NPC cells.

## Highlights

1. Overexpression of Notch2 inhibits the proliferation and enhances the radiosensitivity of NPC cells.

2. Overexpression of Notch2 increases radiation-induced G2/M-phase arrest and apoptosis.

3. Overexpression of Notch2 inhibits the AKT/mTOR signaling pathway in NPC cells.
